# Complete genome of a multiresistant *Pasteurella multocida* isolated from a diseased Austrian bovine calf

**DOI:** 10.1128/mra.00212-24

**Published:** 2024-06-04

**Authors:** Leonard Barta, Beatriz Daza Prieto, Anna Stöger, Daniel Polzer, Friedrich Schmoll, Tatjana Sattler, Werner Ruppitsch

**Affiliations:** 1 Institute for Veterinary Investigations Mödling, Austrian Agency for Health and Food Safety, Mödling, Austria; 2 Institute of Medical Microbiology and Hygiene, Austrian Agency for Health and Food Safety, Vienna, Austria; 3 University of Leipzig, Clinic for Ruminants and Swine, Leipzig, Germany; 4 Department of Biotechnology, University of Natural Resources and Life Sciences, Vienna, Austria; 5 Faculty of Food Technology, Food Safety and Ecology, University of Donja Gorica, Podgorica, Montenegro; University of Maryland School of Medicine, Baltimore, Maryland, USA

**Keywords:** antibiotic resistance, *Pasteurella*, veterinary microbiology, veterinary pathogens

## Abstract

*Pasteurella multocida* (*P. multocida*) plays an important role in bovine respiratory diseases. Here we describe the complete genome of a multiresistant *P. multocida*. The strain was isolated from the lung of a diseased, 4-month old, male Fleckvieh calf with a clinical history of bronchopneumonia in Upper Austria.

## ANNOUNCEMENT

*Pasteurella multocida* can be isolated from the upper respiratory tract of a broad variety of mammals (1). In cattle, *P. multocida* can act as a primary pathogen (2, 3) as well as an opportunistic pathogen in combination with stress or other bacterial or viral agents (4). Due to the ability to obtain various resistance genes (5), correct treatment can be delayed resulting in serious economic consequences and poor animal welfare (6).

*P. multocida* Pm126 was isolated from the lung of a diseased calf in Austria in September 2022. The lung was cut open with a sterile scalpel. Then a sterile swap was taken of the inner part of the organ and was plated onto Columbia agar supplemented with 5% sheep blood (COS, Biomérieux, Marcy-l'Étoile, France) and incubated at 37°C with ambient air for 48 h. Colonies with the appearance of *P.multocida* were subcultured and analyzed *via* MALDI-TOF MS using the MALDI Biotyper Sirius IVD System, MBT Compass HT database v5.1.300 (Bruker Daltonics GmbH & Co. KG, Bremen, Germany) and further characterized by whole-genome sequencing.

The resistance testing was performed with the microdilution system MICRONAUT S Großtiere (Bruker Daltonics GmbH & Co. KG, Bremen, Germany) according to the CLSI protocol (7) using CAMHB (cation adjusted Mueller Hinton Broth, Bruker Daltonics GmbH & Co. KG, Bremen, Germany) supplemented with 2.5% of LHB (leaked horse blood, Thermo Scientific, Hampshire, UK).

Genomic DNA was isolated using the MAGAttract HMW kit (Qiagen, Hilden, Germany) from cells grown overnight on COS at 37°. The Illumina short-read sequencing library was prepared using NexteraXT chemistry and subsequently, 2 × 150 bp sequenced on a NextSeq2000 (Illumina, San Diego, CA, USA).

The DNA library was prepared with the Native Barcoding Kit 24 V14 (SQK-NBD114.24, Oxford Nanopore Technology) without DNA shearing or size selection and sequenced using a FLO-MIN114 (R10.4.1, Oxford Nanopore Technology) SpotON flow cell on a GridION according to the manufacturer’s instructions (Oxford Nanopore Technologies, UK).

A total of 55,897 Nanopore reads, with an average read length of 3,049 bp and one contig were obtained using Dorado v7.1.4 in super-accurate base calling mode and filtered using Filtlong v0.2.1 with the following parameters: min_length, 1,000; keep percent, 90; target_bases, 500,000,000. A total of 2,404,830 Illumina reads were used untrimmed in the final hybrid assembly using Unicycler v0.5.0 (https://github.com/rrwick/Unicycler), which resulted in one contig with a mean coverage of 134-fold, a total length of 2,320,135 bp and a GC content of 40,42 (Table 1). Unicycler v0.5.0 reported the contig as circular.

**TABLE 1:** Characteristics of *Pasteurella multocida* strain Pm126

Parameter	Result
Assembly size (bp)	2,320,135
contig	1
Short reads	2,404,830
Long reads	55,897
Long-read fragment N50 (bp)	8,190
GC content	40.42
Genome coverage	134.8
Genes (total)	2,201
CDs (total)	2,122
tRNA	56
Complete rRNA	7, 6, 6 (5S, 16S, 23S)
Pseudo genes (total)	45
CRISPR arrays	2
ARGs	*aac (3)-IIe* *aph(3')-Ia* *aph(3'')-Ib* *mef(C)* *mph(G)* *catA3* *sul2* *tet(Y*)
MIC gamithromycin	>8 µg/mL
MIC gentamicin	>8 µg/mL
MIC tetracycline	>8 µg/mL
MIC tulathromycin	>64 µg/mL

*P. multocida* strain Pm126 was screened for antimicrobial resistance genes using AMRFinderPlus v3.11.2 (8). Default parameters were used for all software unless otherwise specified. Detected genes are listed in Table 1.

According to the MLST scheme RIRDC from the *P. multocida* PubMLST Database (https://pubmlst.org/organisms/pasteurella-multocida) (9) strain Pm126 belongs to sequence type (ST)79, which is a known virulent type in cattle (10–12).

Approval from the ethics committee was not needed because the project was no animal experiment. The bacterial sample was maintained during routine diagnostic testing and was then further investigated without including the living animal.

## Data Availability

This whole-genome shotgun project has been deposited in the NCBI GenBank database under the accession number CP143079, Bioproject accession number PRJNA1066597, and BioSample accession number SAMN39497375. This is the first version of this genome. The raw sequence reads have been deposited in the Sequence Read Archive (SRA) under the accession numbers SRR27639517 and SRR27639531.
